# Collective dynamics of dark web marketplaces

**DOI:** 10.1038/s41598-020-74416-y

**Published:** 2020-11-02

**Authors:** Abeer ElBahrawy, Laura Alessandretti, Leonid Rusnac, Daniel Goldsmith, Alexander Teytelboym, Andrea Baronchelli

**Affiliations:** 1 Department of Mathematics, City, University of London, London, EC1V 0HB UK; 2 Chainalysis Inc, New York, NY USA; 3 Department of Applied Mathematics and Computer Science, Technical University of Denmark, 2800 Kongens Lyngby, Denmark; 4 Copenhagen Center for Social Data Science, University of Copenhagen, 1353 Copenhagen, Denmark; 5 Department of Economics, University of Oxford, Oxford, UK; 6 UCL Centre for Blockchain Technologies, University College London, London, UK; 7 The Alan Turing Institute, British Library, 96 Euston Road, London, NW12DB UK

**Keywords:** Complex networks, Computational science

## Abstract

Dark web marketplaces are websites that facilitate trade in illicit goods, mainly using Bitcoin. Since dark web marketplaces are unregulated, they do not offer any user protection, so police raids and scams regularly cause large losses to marketplace participants. However, the uncertainty has not prevented the proliferation of dark web marketplaces. Here, we investigate how the dark web marketplace ecosystem reorganises itself following marketplace closures. We analyse 24 separate episodes of unexpected marketplace closure by inspecting 133 million Bitcoin transactions among 38 million users. We focus on “migrating users” who move their trading activity to a different marketplace after a closure. We find that most migrating users continue their trading activity on a single coexisting marketplace, typically the one with the highest trading volume. User migration is swift and trading volumes of migrating users recover quickly. Thus, although individual marketplaces might appear fragile, coordinated user migration guarantees overall systemic resilience.

## Introduction

Dark web marketplaces (or “dark markets”) are commercial websites which specialise in trading illicit goods. They are accessible via darknets (e.g., Tor) and vary in specialisation, technology, and primary supported language. *Silk Road*, the first modern dark marketplace launched in 2011, limited its sales to drugs while other dark marketplaces allow trading of weapons, fake IDs and stolen credit cards^1,2^. Most marketplaces simply facilitate transactions between buyers and sellers of illicit goods, however some marketplaces act as sellers and sell directly to buyers. Bitcoin is the universally accepted currency (occasionally together with other cryptocurrencies) on every dark marketplace.

Operating outside the law, dark marketplaces do not offer any protection to customers or vendors. This has led to a proliferation of scam sales and marketplace hacks. Furthermore, marketplaces may be unexpectedly closed either by the authorities or by marketplace administrators themselves, causing significant losses to users. For example, *Silk Road* was shut down in 2013 by the FBI^3^ and in the same year *Sheep Marketplace* was closed by its administrator, who vanished with 100 million US dollars stolen from its users^4^. Following these events, dark marketplaces have adopted better technologies to mitigate losses caused by closures and to reassure their customers^5–7^. However, this has not prevented further marketplace closures, either due to police raids or due to scams.

Surprisingly, such uncertainty has not prevented a steady growth in users and revenue of dark marketplaces. As of today, there are at least 38 identified active dark marketplaces^8^. Although it is difficult to identify relevant transactions from the Bitcoin blockchain and to quantify marketplace volume^8–11^, European authorities have estimated that between 2011 and 2015 dark marketplace drug sales were 44 million US dollars per year. A subsequent study estimated that, in early 2016, dark marketplace drug sales have grown to between 170 million and 300 million US dollars per year^12^. Recently, *Berlusconi*, known mostly for selling stolen IDs, was seized by the Italian police who estimated its annual transactions at 2 million euros^2^.

Several papers have attempted to study dark marketplaces. However, the difficulty of identifying relevant transactions^8–11^ has forced researchers to rely mostly on user surveys^13,14^ and data scraped from dark marketplace websites^10,15^ (even though dark marketplace administrators actively fight web scraping which is perceived as a threat). Police shutdowns have been shown to correlate with a sudden increase in drug listings in coexisting marketplaces^16,17^. The most comprehensive study on closures among 12 dark marketplaces concluded “that the effect of law enforcement takedowns is mixed as best”^10^. Another recent analysis of a large 2014 police operation identified an impact of closures on the supply and demand of drugs (but not on their prices)^15^. Recent research on attributing anonymised Bitcoin addresses to named entities^18–21^ has not yet been applied to the investigation of the dynamics of dark marketplaces.

In this paper, we investigate the dynamics of 24 dark marketplace closures by looking at 31 dark marketplaces in the period between June 2011 to July 2019. We do so by analysing a novel dataset of Bitcoin transactions involving dark marketplaces assembled on the basis of the most recent identification methods^22–24^. We are therefore able to quantify the overall activity of the major dark marketplaces, in terms of the number of users and the total volume traded. We show that the closure of a dark marketplace, due to a police raid or an exit scam, has only a temporary effect on trading volumes, suggesting that dark marketplace ecosystem is resilient. We provide the first systematic investigation of dark marketplace user migration following an unexpected closure, and show that closures mainly affect low activity users while high activity users migrate quickly to a new marketplace. Finally, we reveal a striking pattern of post-closure coordination: 66% of migrating users choose to move their activity to the same coexisting marketplace. Moreover, the marketplace that receives the largest number of migrating users tends to have the largest volume and the most users in common with the closed marketplace.

## Methods

Dark marketplaces operate similarly to other online marketplaces, such as *eBay*, *Gumtree* or *Craigslist*, on which vendors advertise their products and prices. Customers request shipment through the website and vendors are usually responsible for delivery. Typically, transactions flow from buyers to the dark marketplace which then sends the money to sellers after buyers confirm the receipt of the goods. Customers may leave reviews that contribute to vendors’ reputation^7^. Dark marketplaces are also supported by search engines and news websites such as *Grams*, *DeepDotWeb* and *darknetlive* which aggregate information on all active dark marketplaces^25^. Following multiple scams, dark marketplaces have begun to rely on escrow systems. Dark marketplaces do not keep buyers’ Bitcoins in local addresses but instead send them to an escrow service. Escrow services can be independent from the dark marketplace or integrated with the dark marketplace; either way users can withdraw their money (refund it) if the shipment was not delivered. After the buyer’s confirmation of receipt, the escrow service transfers the money to the seller.

Our analysis relies on a novel dataset of dark marketplace transactions on the Bitcoin blockchain. The ledger of Bitcoin transactions (the blockchain) is publicly available and can be retrieved through Bitcoin core^26^ or a third-party APIs such as *Blockchain.com*^27^. It consists of the entire list of transaction records, including time, transferred amount, origin and destination addresses. Addresses are identifiers of 26–35 alphanumeric characters that can be generated at no cost by any Bitcoin user. Therefore, a single Bitcoin wallet can be associated to multiple addresses. Even though it is called a Bitcoin “wallet”, it is better described as a “keychain” (similar to the macOS Keychain): users have multiple keys which allow them to access multiple addresses where their Bitcoins are stored. In fact, to ensure privacy and security, most Bitcoin software and websites help users generate a new address for each transaction. In order to be useful, therefore, blockchain data has to be pre-processed to map groups of addresses to individual users.

We used data pre-processed by Chainalysis Inc. following approaches detailed in^22–24^. The pre-processing relies on state-of-the-art heuristics^18–21,28^, including cospending clustering, intelligence-based clustering, behavioural clustering, and entity identification through direct interaction^23^. These techniques rely on the observation of patterns in the Bitcoin protocol transactions and user behaviour. First, addresses were grouped based on a set of conditions, following some of the heuristics mentioned above and discussed in Supplementary Information Section S1. Addresses meeting all conditions were included as part of a single cluster. Note that this step is unsupervised and there is no ground truth regarding the mapping between addresses and entities^20^. Then, clusters were identified as specific dark marketplaces, using transaction data collected by Chainalysis Inc. (the technique employed for the identification is similar to the one described in^20^). Identification of addresses by Chainalysis Inc. related to illicit activities has been relied upon in many law enforcement investigations^29,30^. Given the potential uses of identified Bitcoin data, rigorous investigation and avoidance of false positives is crucial. If an address does not meet all the conditions required by the clustering and identification heuristics, it will be tagged as “unnamed”. This means that some addresses belonging to a dark marketplace administrator or dark marketplace users are not included in our dataset (see more information on our dataset in Supplementary Information Section S1).

**Figure 1:**
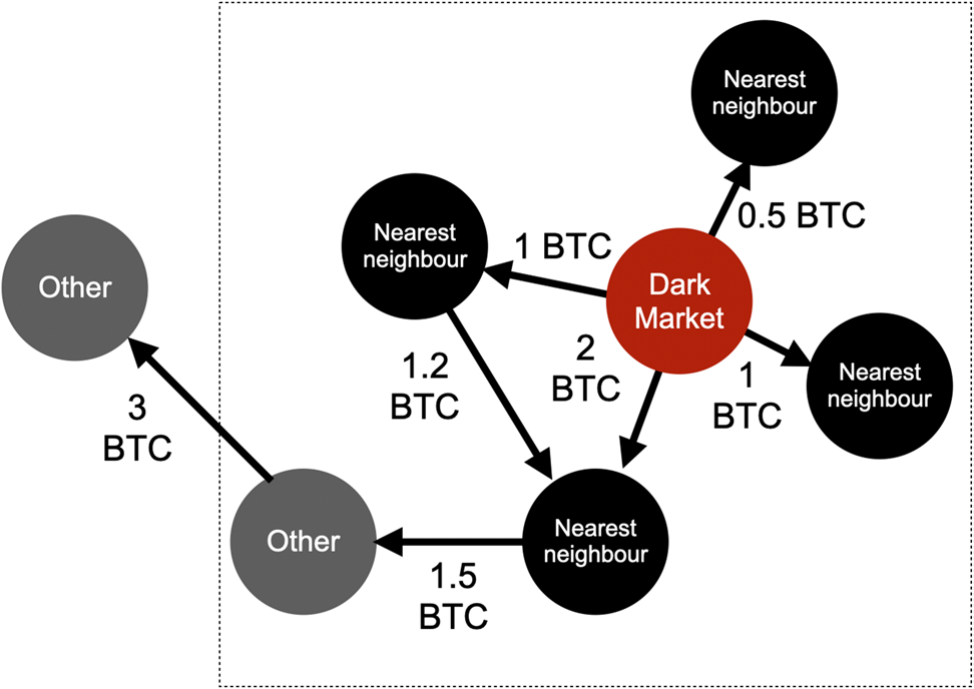
Dark marketplace ego-network. Our dataset includes transactions between addresses belonging to a dark marketplace (in red) and its nearest neighbours (in black), as well as the transactions between nearest neighbours and “other” Bitcoin addresses (in grey). Arrows correspond to transactions, and their value in Bitcoin (BTC) is reported. Any transaction between two “other” nodes is excluded from our dataset. In this schematic representation, the dotted square includes transactions included in our dataset.

We considered the entire transaction data of 31 dark marketplaces (see Supplementary Information Section S2) between June 18, 2011, and July 24, 2019. This dataset includes all the major marketplaces on the darknet as identified by the reports of law enforcement agencies^3,31^ and the World Health Organization^32^. We also considered transactions of all users who interacted with one of these marketplaces (dark marketplace’s “nearest neighbours”) after their first interaction with a dark marketplace. Thus, each dark marketplace can be represented as an egocentric network^33^ of radius 2, where the marketplace is the central node, its nearest neighbours represent marketplace users, and “other nodes” appear only through their interaction with one of the marketplaces users. A direct edge represents a transaction occurring either between the dark marketplace and one of its nearest neighbours, or between two nearest neighbours, or between the nearest neighbour and some “other node”. We excluded Bitcoin trading exchanges from our list of nearest neighbours since we focus on the users’ direct interaction with the dark marketplace. (Bitcoin trading exchanges are platforms that allow users to trade Bitcoin for other cryptocurrencies or fiat currencies.) Figure 1 shows a schematic representation of our dataset, where transactions within the square are the ones included in the dataset. After removing transactions to/from Bitcoin trading exchanges, the dataset contains $\sim 133$ million transactions among over 38 million distinct users. The total number of addresses which directly interacted with dark marketplaces is $\sim 8.3$ million. The volume of transactions sent and received by dark marketplace addresses amounts to $\sim 4.2$ billion US dollars.

**Figure 2:**
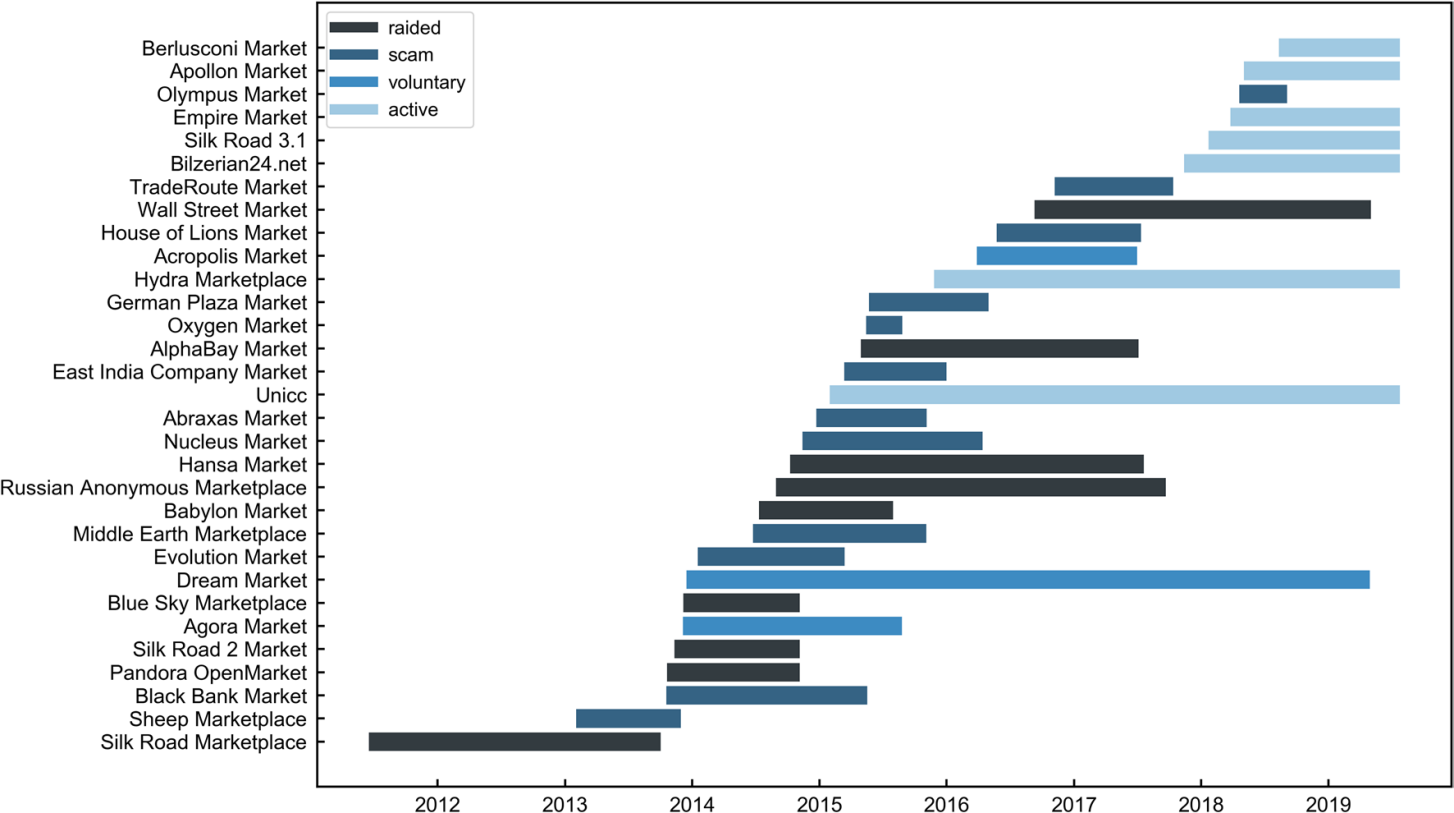
Dark marketplace lifetimes. Each bar corresponds to a different dark marketplace (see y-axis labels). Bars are coloured according to the reason behind closure: raided by the police (black), exit scam (dark blue), voluntary closure (blue). Light blue bars correspond to marketplaces that are still active in November 2019.

In order to gain information on the analysed marketplaces, we collected additional data from the *Gwern* archive on dark marketplace closures^1^. To compile comprehensive information, we also used law enforcement documents on closures as well as a number of online forums^31,32,34^ dedicated to discussing dark marketplaces (see Supplementary Information Section S2). Out of the selected marketplaces, 12 were subject to an exit scam, 9 were raided, 3 were voluntarily closed by their administrators, and 7 are still active. 29 marketplaces operate in English and 2 operate in Russian. Out of the 31 marketplaces, 3 are marketplaces dedicated to fake and stolen IDs and credit cards. The primary currency on these marketplaces is Bitcoin. In Fig. 2, we present the lifetimes of the selected marketplaces and the reasons behind their closures.

## Results

The dataset contains 133,308,118 transactions among 38,886,758 users. The total number of distinct users which directly interacted with a dark marketplace is 8,377,478. The volume of transactions sent and received by dark marketplace addresses amounts to 4.210 billion US dollars, while the volume received by dark marketplaces addresses is 1.99 billion US dollars. Note that the conversion between Bitcoins and US dollars is done using the price of Bitcoin at the time of the transaction. Table S2 in Supplementary Information Section S2 reports characteristics of the 31 marketplaces analysed in this paper, including the overall number of users and transaction volume. The most active marketplace in terms of number of users and traded volume is *AlphaBay*, followed by *Hydra*.

### Resilience of the dark marketplace ecosystem

**Figure 3:**
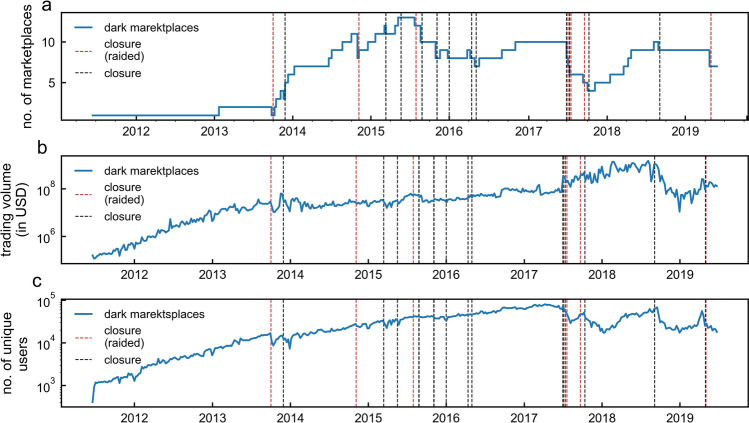
Resilience of the dark marketplace ecosystem. (**a**) The total number of active dark marketplaces across time. (**b**) The total volume (in US dollars, USD) exchanged by dark marketplace addresses. (**c**) The number of unique users interacting with dark marketplaces. Dashed lines represent marketplace closures due to law enforcement raids (in red), or any other reason (in black). Values are calculated using a time window of 1 week.

The capacity of the dark marketplace ecosystem to recover following a marketplace closure can be studied by quantifying the evolution of the total volume traded by dark marketplaces over time. Despite recurrent closures, we find that the number of dark marketplaces has been relatively stable since 2014 as new dark marketplaces frequently open (see Fig. 3a). In addition, despite closures, the total weekly volume sent/received by dark marketplace addresses has been growing steadily between 2014 and the end of 2019 (see Fig. 3b). In fact, Moving Average Convergence Divergence (MACD) analysis^35^ reveals that, following each dark marketplace closure, the overall dark marketplace volume drops, but it recovers quickly thereafter, within 9 days on average (median: 3 days, see also Figure S6 in Supplementary Information). Starting from the end of 2018, however, we observe a decrease in the total volume traded (See Fig. 1).

### User migration

The observation that trading volumes recover quickly after unexpected marketplace closures suggests that users may move to other dark marketplaces^15,36^. We refer to this phenomenon as *migration*.

In fact, migration was observed^37^ after the closure of the *AlphaBay* marketplace when other marketplaces, namely *Hansa* and *Dream Market*, experienced an abnormal spike in activity. In this section, we provide the first systematic investigation of dark marketplace user migration, by studying the effects of a series of closures.

**Figure 4:**
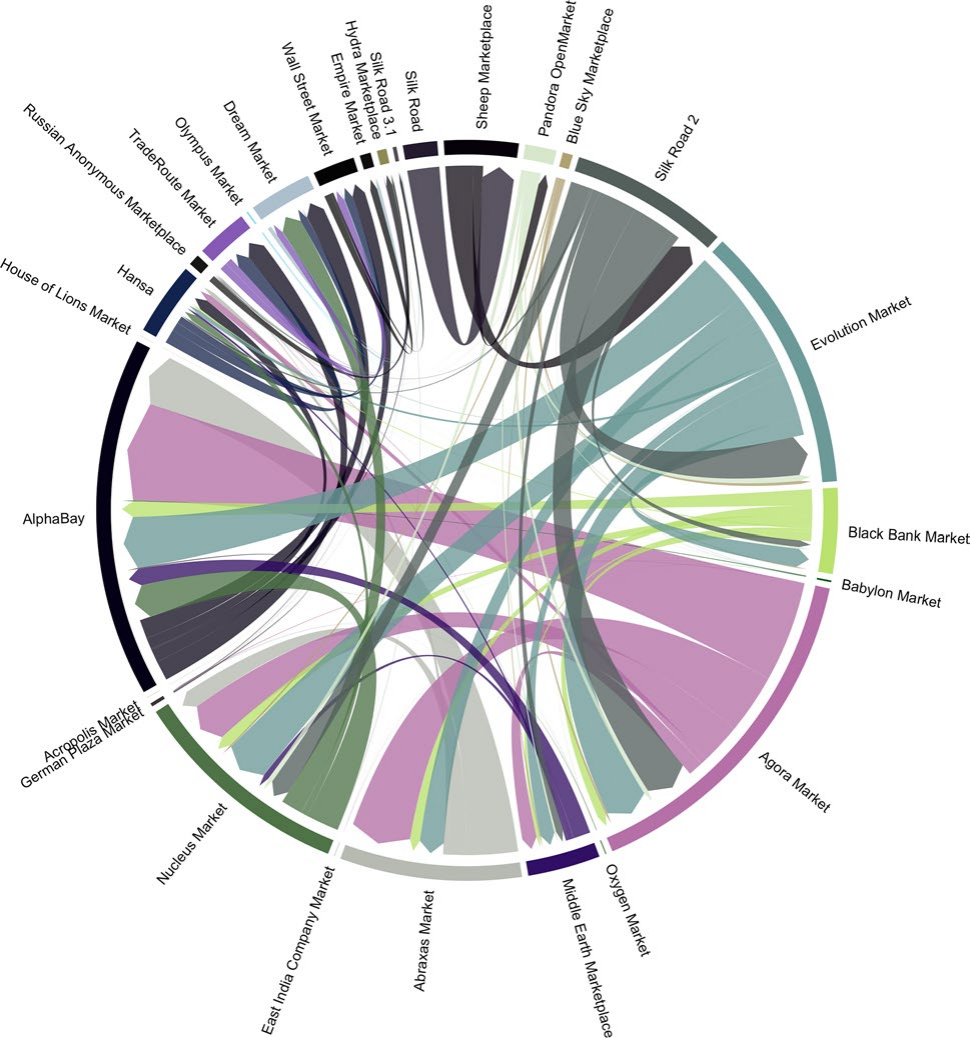
Migration of users following dark marketplace closures. Flows of users migrating to another coexisting marketplace following a closure. The arrowhead points to the direction of migration, and the width of the arrow represents the number of users. Marketplaces are ordered clockwise according to their closure dates in ascending order starting from *Silk Road*.

We identify “migrant users” in the following way. For each dark marketplace *m* that was shut down, we identify users who started trading on another coexisting marketplace $m'$ after the closure of *m*. If a user was trading on both marketplaces *m* and $m'$ before the closure of marketplace *m*, the user is not labelled as a migrant to marketplace $m'$. Figure 4 shows the flows of migrant users between marketplaces.

But what is the fraction of users that migrates after a closure? To answer this question we need to take into account the fact that $\sim 38\%$ of all users in our dataset made only one transaction (sent or a received once)—a finding consistent with the evidence that most of the minted Bitcoins were accumulated in addresses which never sent them, at least until 2013^19^). We then need to estimate the expected number of users who would have kept transacting with the dark marketplace if no closure had occurred. To do so, we focus on the “returning users” over time, i.e., the fraction of all users active (i.e., who sent or received Bitcoins) in a given week that are also active on the following day. We denote as $R_t$ the intersection between the set of active users on a given day *t*, and the set of users that were active at least once in the preceding week. Thus, $R_t$ is the set of “returning users”. In order to compare the number of returning users across closures, we normalise the entire time series by the fraction of returning users at the time of closure, $R_{\hat{t}}$ where $\hat{t}$ is the day of closure. Thus, the normalised value of returning users on the day of closure is 1. Then, we consider the median normalised value across marketplace closures. We find that, 5 days after the closure of a dark marketplace,  the median normalised value of returning users across market closures is .85. This finding indicates that, even though marketplace closure affects participation, the vast majority of returning users migrate to another dark  marketplace following a closure.

### Who is migrating?

**Figure 5:**
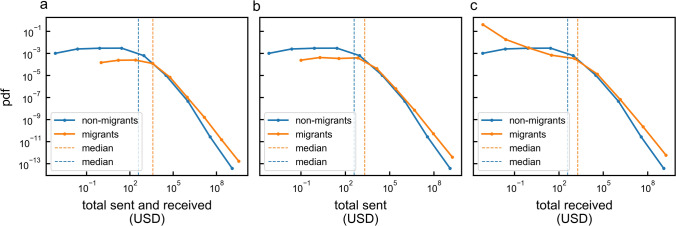
Migrant user versus non-migrant user activity distribution. (**a**) The distribution of the total volume sent and received across all closed dark marketplaces for migrants (orange line) and non-migrants (blue line). (**b**) The distribution of the total volume sent across all closed dark marketplaces by migrants (orange line) and non-migrants (blue line). (**c**) The distribution of the total volume received across all closed dark marketplaces by migrants (orange line) and non-migrants (blue line). Dashed lines represent the median value for migrants (orange line) and non-migrants (blue line).

The observation that some users stop trading following a dark marketplace closure but the total volume traded in dark marketplaces does not decrease could indicate that migrant users are on average more active than others. We test this hypothesis by computing the activity of migrant users before and after dark marketplace closure. We refer to the original dark marketplace that a user was interacting with as its “home marketplace”. For all users (migrant and non-migrant), we measure the total volume exchanged with any other user in our dataset including the home marketplace. We find that the median volume exchanged by migrant users is $\sim 10$ times larger than the volume exchanged by non-migrant users (see Fig. 5a), with the median volume exchanged summing to 3882.9 US dollars across all migrant users and to 387.2 US dollars for non-migrant users. The means are sensitive to high volume users, with 71,6441.9 US dollars and 17,529.7 US dollars for migrant and non-migrant users respectively (see Fig. 5). In terms of receiving and sending behaviour, migrants users are also more active compared to non-migrants (see Fig. 5b,c). Similar conclusions can be drawn by considering the volume exchanged with the home marketplace only, with median values of 263 US dollars for migrant users and 74.3 US dollars for non-migrants users and mean values of 2725.1 US dollars and 475.9 US dollars for migrant and non-migrant users respectively (see Supplementary Information Section S4).

The activity distribution of migrants is significantly different from the non-migrant users’ distribution (using Kolmogorov–Smirnov test, $p < 0.01$, see Table S3 in Supplementary Information Section S4).

### Coordination in the dark

We now turn to the analysis of how migrant users decide where to migrate. In our dataset, following every instance of a marketplace closure except one, users could migrate to two or more coexisting marketplaces.

**Figure 6:**
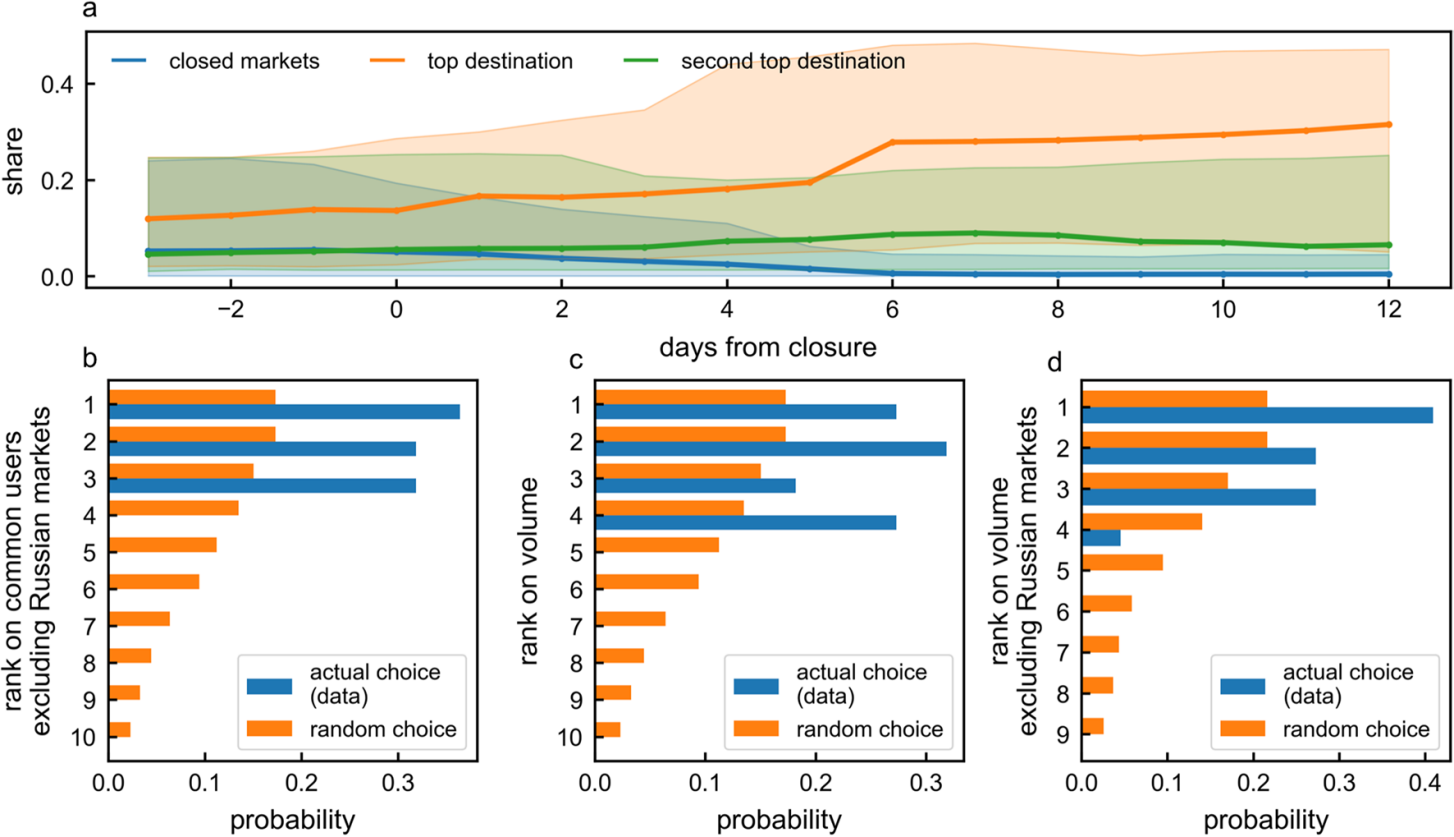
Migration decision and impact. (**a**) The median share (across closures) of a closed marketplace (blue line), the top destination marketplace for the migrant users (orange line) and the second top destination for migrant users (green line). The shaded area represents the 50% interquartile range. Values are computed using a rolling window of 1 week. (**b–d**) show the probability of a marketplace being chosen for migration (becoming the top destination for migration) given its rank at the time of coexisting marketplace closure compared to the random model. Marketplaces are ranked in descending order according to (**b**) the number of overlapping users they have with the closed marketplaces excluding Russian marketplaces (**c**) the total trading volume in US dollars and (**d**) the total trading volume in US dollars excluding Russian marketplaces from the ranking. The random model in (**b**–**d)** represents a model where users migrate to any coexisting marketplace with equal probability.

In Fig. 6a, we show the evolution of the trading volume shares of closed marketplaces and the top two destination marketplaces in the days preceding and following a closure. Trading volume share for a given market is the trading volume of a market normalised by the total trading volume of all dark marketplaces. We find that the top two destination marketplaces experience an increase in the trading volume share starting 2 days after the closure, and saturating about 6 days after with a share of $27\%$, more than double the share at the time of closure. The second top destination, on the other hand, increases its share from 5 to 8.7%.

We investigate the characteristics of the top destination marketplace for migrant users, by ranking coexisting marketplaces according to the total trading volume in US dollars at the time of closure and the total number of common users between the closed and the coexisting marketplace before closure. We find that, regardless of the reason behind closure, users do not migrate randomly, but rather  choose to move to the marketplace with the highest trading volume which, in some cases, is also the marketplace with the highest number of common users.

Focusing on the first week after closure, we find that, on average, one marketplace absorbs $66.1\% \pm 16.1$ of all migrant users. Only 4% of the users migrate to more than one coexisting marketplace simultaneously after the closure. What kind of marketplace is this? Figure 6b shows that, in 36.4% of the closures considered, it is the one sharing the largest number of common users with the closed marketplace(the probability that users migrate to the marketplace ranked second or the third is 31.8%). Users do not choose to migrate to marketplaces that have fewer common users than the third-ranked marketplace.

Figure 6c shows that, when marketplaces are ranked according to transaction volume, the second-largest marketplace is preferred in the majority of cases (31.8%). However, a closer look at the data reveals that a Russian marketplace often occupies the top ranks in terms of volume but it tends not to be the preferred migration harbour, probably due language and geographical barriers. Excluding the Russian marketplace from the ranking, we find that, in fact, the largest marketplace by volume is selected 41% of the time (see Fig. 6d).

We compare the users’ decisions with a null random model, where after each closure users move with equal probability to any of the existent marketplaces. The probability $P_i$ of the $i{\text {th}}$-ranked marketplace to be chosen for migration uniformly at random after *m* closures is equal to

$$\begin{aligned} P_i =\frac{\sum ^{m}_{j=1}{1/c_j}}{m}, \end{aligned}$$

where $c_j$ is the number of coexisting marketplaces at the time of closure of *j*. We find that the data differ significantly from the uniform random choice model, confirming the presence of coordination between migrating users (see Fig. 6).

## Discussion

We analysed a novel dataset of Bitcoin transactions on 31 major dark marketplaces and investigated how the dark marketplace ecosystem was affected by unexpected marketplace closures between 2013 and 2019. The dark marketplaces we considered were heterogeneous in many ways and 24 of them were closed abruptly due to police raids and scams. We found that the total volume traded on these dark marketplaces dropped only temporarily following closures, revealing a remarkable resilience of the marketplace ecosystem. We identified the origin of this resilience, by focusing on individual users, and unveiled a swift and ubiquitous phenomenon of migration between recently closed and coexisting marketplaces. We found that migrating users were more active in terms of total transaction volume compared to users who did not migrate. Finally, we found that migrating users tended to migrate predictably to coexisting dark marketplaces which had the largest overall volumes and the highest numbers of users in common with the closed marketplaces.

Our findings shed a new light on the consequences of sudden closure and/or police raids on dark marketplaces, which have been previously  discussed in the literature and analysed by law enforcement entities^12,15,31^. Interesting future research directions include the role of dark marketplace closure on the emergence of new marketplaces, refining the analysis to investigate whether scam closures and police raids may have had other effects on user migration, delving deeper into the types of user behaviour that can predict migration, and broadening the research to include the effect of online forums on the performance of existing marketplaces as well as on the migration choices after a closure^34^. More broadly, we anticipate that our findings will help inform future research on the self-organisation of emerging online marketplaces.

## Supplementary information

Supplementary Information.

## Data Availability

All data needed to evaluate the conclusions in the paper are present in the paper. Additional data related to this paper may be requested from the authors.
