# Supplementary material for: Collective dynamics of dark web marketplaces
Source: Sci Rep. 2020 Nov 2;10:18827. doi: 10.1038/s41598-020-74416-y (PMC7608591; doi:10.1038/s41598-020-74416-y)
Supplement: Supplementary file 1 — Supplementary Information. [file 41598_2020_74416_MOESM1_ESM.pdf]

Supplementary Information for: Collective Dynamics of Dark Web Marketplaces

Abeer ElBahrawy[a,b], Laura Alessandretti[c], Leonid Rusnac[b], Daniel Goldsmith[b], Alexander Teytelboym[d], and Andrea Baronchelli[a,e,f,*]

[a]City, University of London, Department of Mathematics, London EC1V 0HB, UK

[b]Chainalysis Inc, NY, USA

[c]Technical University of Denmark, DK-2800 Kgs. Lyngby, Denmark

[d]Department of Economics, University of Oxford, UK

[e]UCL Centre for Blockchain Technologies, University College London, UK

[f]The Alan Turing Institute, British Library, 96 Euston Road, London NW12DB, UK

[*]Corresponding author: Andrea.Baronchelli.1@city.ac.uk

S1 Data processing techniques

In Bitcoin, multiple addresses can belong to one user; grouping these addresses reduces the complexity of the ledger and Bitcoin anonymity [1]. Clustering techniques rely on how Bitcoin's protocol works, users behaviour on the blockchain, Bitcoin's transaction graph structure and finally, machine learning. Methods relying on Bitcoin's protocol specifically exploit what is known as change addresses: Bitcoins available in an address have to be spent as a whole. Fig. S1 shows an example of a change address. User A's wallet has two addresses, one contains 1BTC and another has 2BTC. User A would like to transfer 0.25BTC to user B, as shown in Fig. S1A. After transferring the 0.25BTC to B, the change (0.75BTC) will not stay in the same address. Bitcoin protocol will create another address, also assigned to A, where the 0.75BTC change will be stored. By observing this pattern, a heuristic technique proposed in [3] suggests that these addresses can be grouped, as they belong to one user.

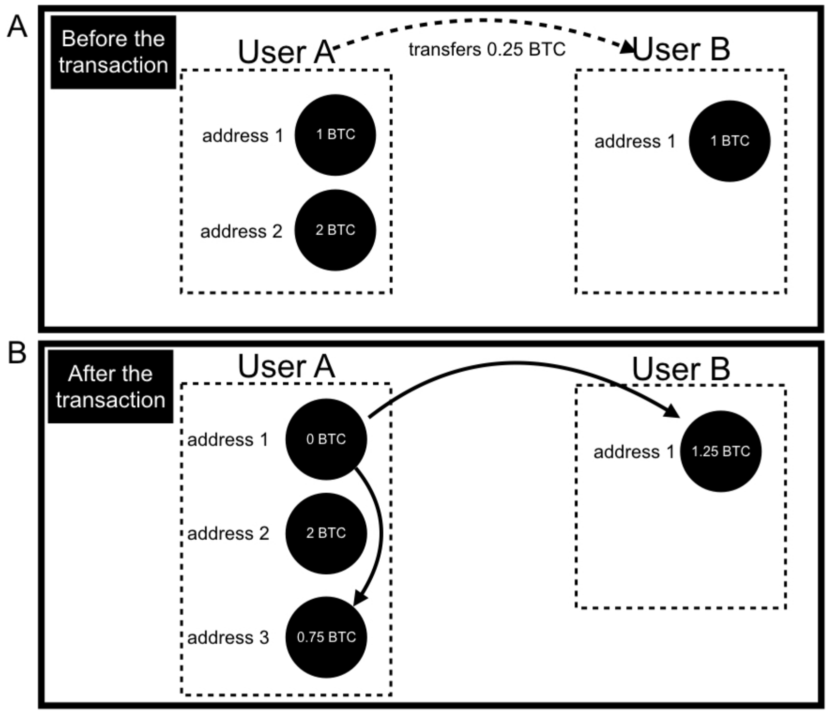

Figure S1: **How Bitcoin's protocol handles transactions with change.** (**A**) A transaction between users A and user B, where A wants to transfer 0.25 Bitcoins to B. User A has two addresses, one with 1 Bitcoin and the other with 2 Bitcoins. User B has one address, containing 1 Bitcoin. (**B**) How a transaction is conducted under Bitcoin protocol. User A first address transfers 0.25 Bitcoin to user B first address. The change of 0.75 Bitcoin does not stay in User A first address 1, but appears, instead, as another transaction to a new address. The dotted boundaries in both figures represent a grouping of these addresses, as they belong to one user. A solid arrow represents an executed Bitcoin transaction, while the dotted arrow represents a desired transaction.

Since users can have multiple addresses, they can use multiple of these addresses to transfer Bitcoins in a single transaction. For example, Fig. S2A shows a case where user A controls 3 different addresses. Each address has a different amount of Bitcoins, 1, 4 and 2.5 respectively. User A wants to transfer 5 Bitcoins to user B, and two addresses will be used to complete the transaction as shown in Fig. S2B. This observation allows the grouping of these two addresses as a single user [3].

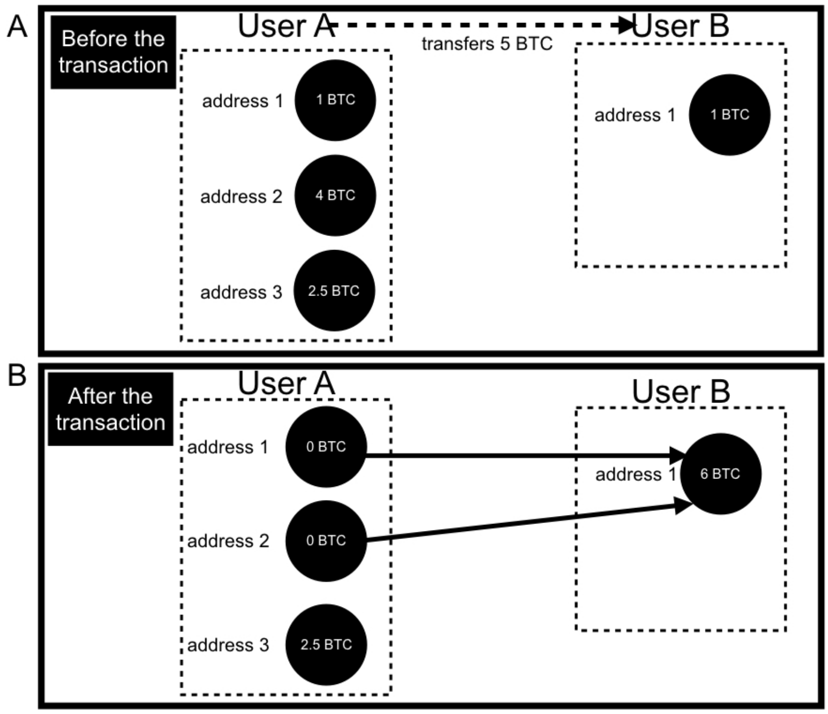

Figure S2: **Sending from multiple inputs in Bitcoin** (**A**) A desired transaction between users A and B, where A wants to send 5 Bitcoins to user B. User A has 3 different addresses with 1, 4 and 2.5 Bitcoins respectively. User B has one address containing 1 Bitcoin. (**B**) How the transaction will be conducted under the Bitcoin protocol. User A will use two addresses to complete the transaction. Both addresses will send to one address belonging to user B. The dotted boundaries in both figures represent a grouping of these addresses as they belong to one user. The solid arrows represent an already executed Bitcoin transaction while the dotted arrow represents a desired transaction.

The work in [2] challenged these heuristics, showing the possibility of having false positives and not taking into consideration changes in the protocol. The work suggests instead a manual process, where the behaviour of each entity is investigated. Page rank (network centrality measure [5]) was also used to identify important addresses [6]; however, the addresses were already grouped using the heuristics introduced by [3].

Mapping addresses to an actual identity is more challenging. Some entities already publish their public key for donation and payment, such as Wikimedia Foundation [7]. The only research that introduced a method for mapping a collection of addresses to a real-world identity is [2], through direct interaction with the address. In this work, researchers directly engaged in 344 transactions with different services including mining pools, exchanges, dark marketplaces and gambling websites.

The introduction of these heuristics did not only challenge Bitcoin's anonymity but also eased the regulation of Bitcoin. Companies specialising in blockchain analytics started to capitalise on these heuristics and provide tools for exchanges and law enforcement entities to facilitate regulatory efforts. For our analysis of dark marketplaces, our data was provided by Chainalysis [8], which is a blockchain analytics company. Chainalysis aided several investigations led by different law enforcement entities, including the United States Internal Revenue Service (IRS) [4].

The dataset we rely on was processed using the state of the art techniques of clustering and identification. For clustering, a set of conditions are composed based on the techniques discussed earlier. If an address meets all the conditions it will be included as part of the cluster. For the clustering process

there is no ground truth dictating that a group of addresses within one cluster are owned by the same entity or not. On the other hand, the identification process relies on actual transactions were conducted similar to [2].

Our dataset sampling approach (from the entire Bitcoin transactions) deploys a complex network perspective. Transactions on the blockchain can be modelled as a directed weighted graph where a node represents a user, and a directed edge between two nodes A and B represents a transaction from user A to user B. Depending on the clustering algorithm, a node can represent one address or multiple addresses. A node can also be labelled as a specific entity or unlabelled (unnamed). Fig. S3 shows a sketch of the network and the different possible meanings of a node. For example in Fig. S3, the black unnamed node on the right side is a representation of two different addresses clustered together, however, they were not attributed to an entity thus remained unnamed.

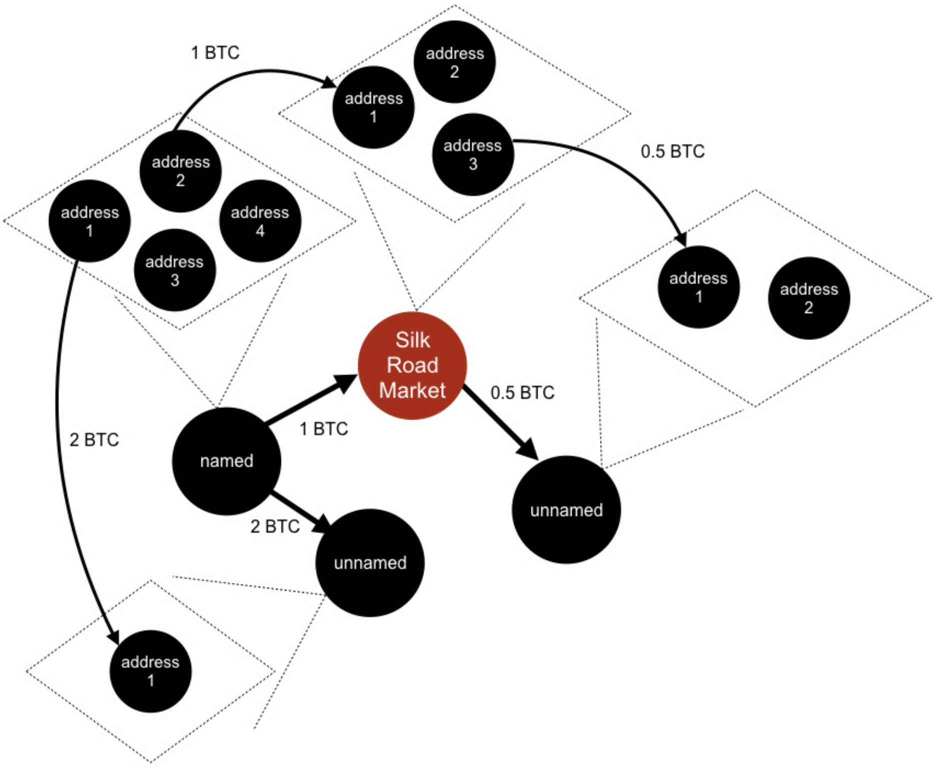

Figure S3: **A dark marketplace's Bitcoin transaction network.** A schematic representation of our dataset as a complex network. Nodes represent users, and a direct edge between two nodes represents a transaction in the direction of the edge. Nodes can represent different abstractions as shown by the dotted rhombus. Starting from the right side, the unnamed black node represents a cluster of two different addresses which, however, was not attributed to a specific entity. The dark marketplace node(in dark red, Silk Road Market), is a representation of 3 addresses and attributed by the algorithm to the marketplace. The black named node on the left side of Silk Road Market node is a representation of 4 addresses and named to belong to a specific entity. Finally, the black unnamed node at the bottom left side of the figure, represents one address.

S2 Dark marketplaces information

In this section we provide data on each marketplace understudy. Table S1 shows general information on the dark marketplaces included in our dataset.

Name	Start date	End date	Closure reason	Sales
Abraxas Market	2014 – 12 – 13	2015 – 11 – 05	scam	drugs
Acropolis Market	2016 – 03 – 27	2017 – 07 – 01	voluntary	mixed
Agora Market	2013 – 12 – 03	2015 – 08 – 26	voluntary	mixed
AlphaBay Market	2014 – 12 – 22	2017 – 07 – 05	raided	mixed
Apollon Market	2018 – 05 – 03	active	active	drugs
Babylon Market	2014 – 07 – 11	2015 – 07 – 31	raided	drugs
Berlusconi Market	2018 – 08 – 12	active	active	mixed
Bilzerian24.net	2017 – 11 – 13	active	active	credits
Black Bank Market	2014 – 02 – 05	2015 – 05 – 18	scam	mixed
Blue Sky Marketplace	2013 – 12 – 03	2014 – 11 – 05	raided	drugs
Dream Market	2016 – 03 – 19	2019 – 04 – 30	voluntary	mixed
East India Company Market	2015 – 04 – 28	2016 – 01 – 01	scam	drugs
Empire Market	2018 – 02 – 01	active	active	mixed
Evolution Market	2014 – 01 – 14	2015 – 03 – 14	scam	drugs
German Plaza Market	2015 – 05 – 22	2016 – 05 – 01	scam	mixed
Hansa Market	2014 – 03 – 09	2017 – 07 – 20	raided	drugs
House of Lions Market	2016 – 05 – 23	2017 – 07 – 12	raided	drugs
Hydra Marketplace	2015 – 11 – 25	active	active	mixed
Middle Earth Marketplace	2014 – 06 – 22	2015 – 11 – 04	scam	mixed
Nucleus Market	2014 – 10 – 24	2016 – 04 – 13	scam	mixed
Olympus Market	2018 – 04 – 20	2018 – 09 – 04	scam	mixed
Oxygen Market	2015 – 04 – 16	2015 – 08 – 27	scam	drugs
Pandora OpenMarket	2013 – 10 – 20	2014 – 11 – 05	raided	drugs
Russian Anonymous Marketplace	2014 – 08 – 29	2017 – 09 – 21	raided	mixed
Sheep Marketplace	2013 – 02 – 28	2013 – 11 – 29	scam	drugs
Silk Road Marketplace	2011 – 01 – 31	2013 – 10 – 02	raided	mixed
Silk Road 2 Market	2013 – 11 – 06	2014 – 11 – 05	raided	mixed
Silk Road 3.1	2018 – 01 – 21	active	active	drugs
TradeRoute Market	2016 – 11 – 06	2017 – 10 – 12	scam	mixed
Unicc	2015 – 01 – 30	active	active	credits
Wall Street Market	2016 – 09 – 09	2019 – 05 – 02	raided	mixed

Table S1: **Dark marketplaces information.** Information on the 31 selected dark marketplaces included in our dataset. For each marketplace, the table states the name of the marketplace, the start and end dates of its operation, the closure reason (if applicable) and the type of products sold by the marketplace. “Drugs” indicates that the primary products sold on the marketplace are drugs while “credits” indicates the marketplace specialises in fake IDs and credit cards and “mixed” indicates the marketplace sells both types of products

Table S2 shows the total volume received and sent by the different marketplaces, as well as the number of their users.

Figure S4 shows the seasonal patterns in Silk Road Marketplace total trading volume in US dollars, which was observed also in other markets and similar behaviour was reported in the literature for Bitcoin overall payment network [**?**]. The seasonal decomposition was done using an additive model and moving averages for the daily time series, 3 days aggregation and weekly trading volume.

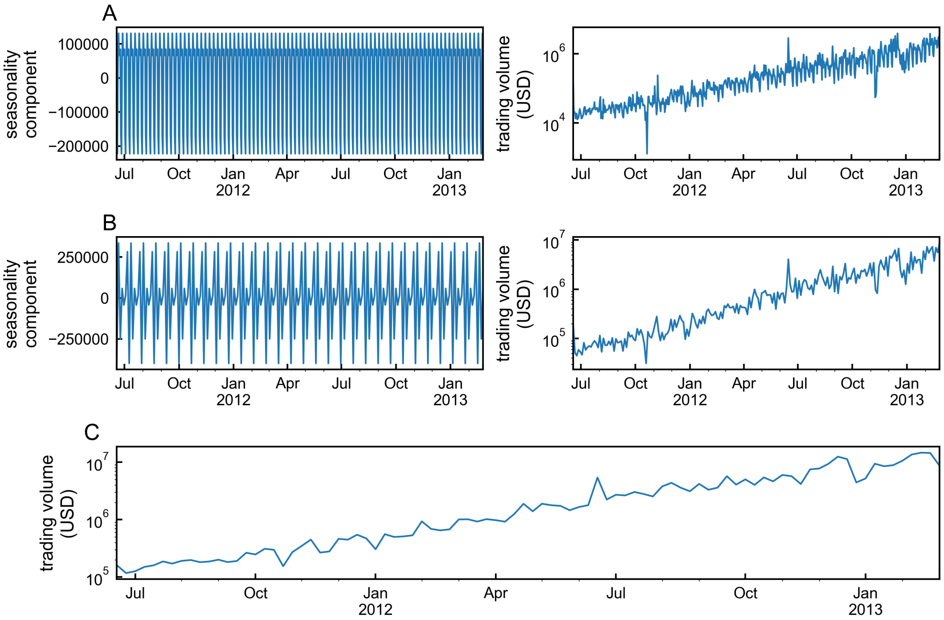

Figure S4: **Silk Road Marketplace seasonality (A)** The right hand side figure shows Silk Road Marketplace daily trading volume and the left hand side figure shows the seasonality observed in the data. **(B)** The right hand side figure shows Silk Road Marketplace trading volume using 3 days time window and the left hand side figure shows the seasonality observed in the data on the right hand side. **(C)** Silk Road Marketplace weekly trading volume which does not exhibit seasonality.

S3 Moving Average Convergence Divergence Analysis

To further quantify the changes in dark marketplaces traded volume, we calculate the Moving Average Convergence Divergence (MACD) of the weekly trading volume. The MACD is a trading indicator used in stock marketplaces to quantify price movements and fluctuations. It is composed of three time series. Firstly, the MACD, calculated as the difference between the exponential weighted moving average of the trading volume for a period of 12 weeks and the exponential weighted moving average of the trading volume for a period of 26 weeks. Secondly, the signal line, computed as the 9 weeks exponential weighted moving average of the MACD time series. Finally, the last time series, known as the histogram, representing the difference between the MACD and the signal line.

Fig. S5 shows the indicator behaviour across time. For each closure, there is a fluctuation in the MACD line and the histogram line indicates a downward change in the overall dark marketplaces volume. However, an upward change can be observed after the closures indicating that dark marketplaces recover.

Name	Volume sent (US dollars)	Volume received (US dollars)	out degree	in degree	Volume tot(US dollars)
Abraxas Market	29, 822, 178.9	23, 044, 463.2	21953	96612	52, 866, 642.1
Acropolis Market	11, 196.7	11, 407.6	101	201	22, 604.3
Agora Market	163, 946, 119.7	148, 224, 155.3	122582	468708	312, 170, 3
AlphaBay Market	605, 445, 951.5	529, 077, 614	267818	1590672	1, 134, 523, 565.2
Apollon Market	17, 384.5	15, 113.6	57	138	32, 498.1
Babylon Market	144, 292.6	149, 257.5	902	1398	293, 550.1
Berlusconi Market	230, 036.6	239, 430.9	514	2153	469, 467.5
Bilzerian24.net	22, 821, 289.6	19, 130, 767.5	108	240232	41, 952, 057.1
Black Bank Market	14, 841, 938.8	13, 858, 325.9	15805	53260	28, 700, 264.8
Blue Sky Marketplace	4, 294, 944.4	3, 297, 912.5	10210	16275	7, 592, 856.9
Dream Market	78, 031, 896.0	60, 049, 434.3	46648	475260	138, 081, 330.3
East India Company Market	3, 638, 096.5	2, 942, 049.9	4630	1951	6, 580, 146.4
Empire Market	11, 962, 986.2	8, 975, 257.2	1309	66124	20, 938, 243.4
Evolution Market	55, 982, 302.9	49, 622, 433.1	35415	219491	105, 604, 735.9
German Plaza Market	1, 032, 802.5	951, 757.3	22	10824	1, 984, 559.9
Hansa Market	62, 087, 671.5	61, 171, 541	73496	336045	123, 259, 212.5
House of Lions Market	705.7	1, 018.4	12	97	1, 724.1
Hydra Marketplace	426, 946, 433.7	474, 549, 308.6	113878	1081883	901, 495, 742.3
Middle Earth Marketplace	9, 861, 173.8	8, 549, 901.3	9503	38506	18, 411, 075
Nucleus Market	70, 112, 730.6	58, 544, 889.4	55522	207791	128, 657, 619.9
Olympus Market	828, 076.9	711, 202.93	1877	4230	1, 539, 279.9
Oxygen Market	42, 914.2	37, 273.5	278	605	80, 187.7
Pandora OpenMarket	9, 422, 325.0	8, 568, 086.9	8864	35859	17, 990, 411.9
Russian Anonymous Marketpl.	131, 000, 457.9	105, 804, 257.1	36794	745939	236, 804, 714.9
Sheep Marketplace	15, 624, 992.4	11, 624, 434.9	7718	38612	27, 249, 427.4
Silk Road 2 Market	85, 610, 718.5	70, 325, 928.9	48293	227239	155, 936, 647.4
Silk Road 3.1	13, 310, 738.1	9, 547, 696.8	15574	64205	22, 858, 434.9
Silk Road Marketplace	172, 812, 766.4	140, 579, 172.6	73114	400079	313, 391, 938.9
TradeRoute Market	18, 313, 990.6	17, 190, 084.7	14318	104413	35, 504, 075.3
Unicc	147, 418, 817.2	106, 581, 024.9	443	1301371	253, 999, 842.1
Wall Street Market	68, 596, 630.4	52, 623, 050.2	26522	359656	121, 219, 680.6

Table S2: **Dark marketplaces overall activity.** The activity of the dark marketplaces as observed in our dataset. For each marketplace, the table reports the total volume sent and received by dark marketplace addresses. It also reports the total number of users who sent (in-degree) and received (out-degree) Bitcoins to/from dark marketplace addresses.

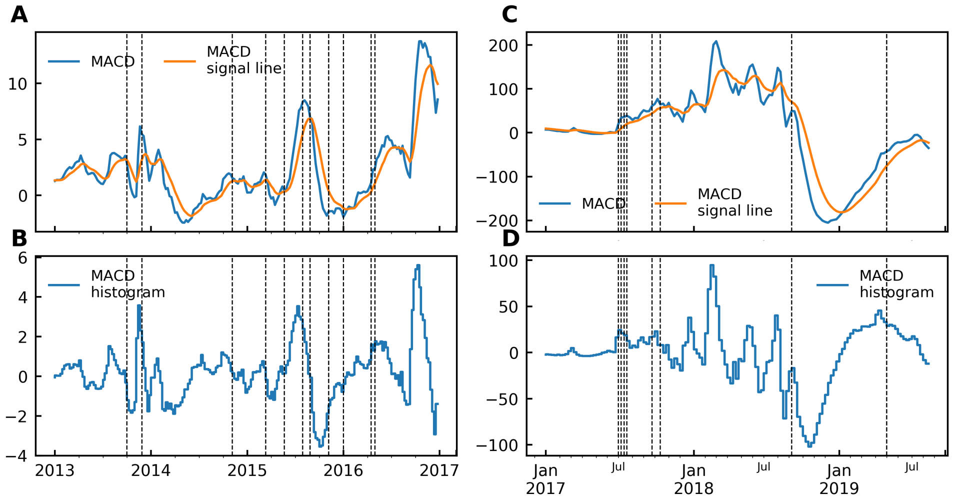

Figure S5: **Moving Average Convergence Divergence (MACD) (A)** The MACD (blue line) and MACD signal line (orange line) for dark marketplaces trading volume from 2013 till 2016. **(B)** The MACD histogram (blue line) for the dark marketplaces trading volume from 2013 to the end of 2016. **(C)** The MACD (blue line) and MACD (orange line) signal line for dark marketplaces trading volume from 2017 until July, 2019. **(D)** The MACD histogram (blue line) for the dark marketplaces trading volume from 2017 until July, 2019. Vertical dashed lines represent marketplaces closure.

Finally, we show in figure S6 the time it took for dark marketplaces' total trading volume to recover after each closure. After the majority of the closures (75% closures) the darknet marketplaces recovered in less than 10 days. Silk Road Marketplace had the longest rebound time of 69 days. Note that also there were compound closures where two markets closed at the same time which we consider here as one closure event and we measure the rebound afterwards. Also at the end of the covered period a market closure occurs.

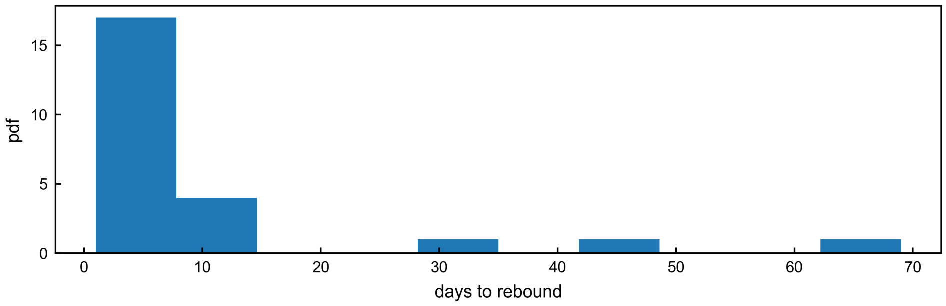

Figure S6: **Time till dark marketplaces's trading volume recovered** The distribution of the number of days it took dark marketplaces trading volume to recover after each closure.

S4 Migrant and non migrants

In the main text we show that for each closed marketplace, migrant users are more active in terms of the total amount they send and received overall, specifically with the closed dark marketplace. In this section, we show the behaviour across each closed marketplaces. Fig. S7 shows that activity for migrants overall is higher than the non-migrants for each closed marketplace.

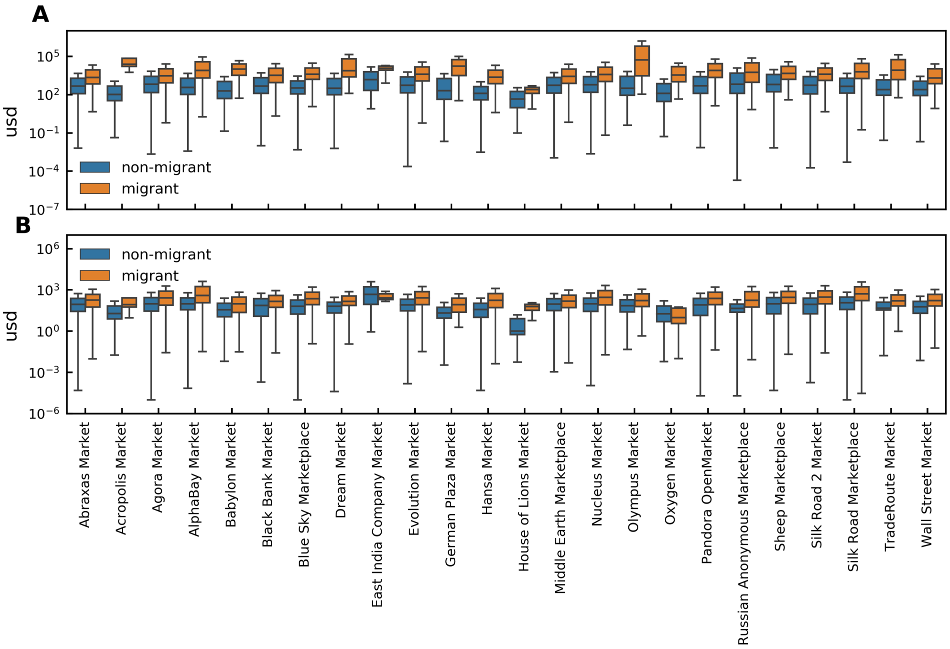

Figure S7: **Migrants are more active than other users.** **(A)** Total volume exchanged by migrant users (orange box-plots) and non-migrant users (blue box-plots) before the closure of their home marketplace. **(B)** Volume exchanged by migrant users (orange box-plots) and non-migrant users (blue box-plots) with their home marketplace. The horizontal line in each box represents the median. The lower box boundary shows the first quartile, and the upper one shows the third quartile. The whiskers show the minimum and maximum values within the 1.5 lower and upper interquartile range.

Table S3 shows the results of a Kolmogorov smirnov test between the migrant and non migrant activity distribution.

**Dark marketplace**	***p*-value (dark marketplace transactions)**	***p*-value (all transactions)**
Abraxas Market	$5.9 * 10^{-85}$	$9.5673 * 10^{2}43$
Agora Market	0	0
AlphaBay Market	0	0
Babylon Market	$3.794 * 10^{-04}$	$8.161 * 10^{-17}$
Black Bank Market	$1.632283 * 10^{-42}$	$1.735524 * 10^{-159}$
Blue Sky Marketplace	$1.138519 * 10^{-22}$	$6.731465 * 10^{-67}$
Dream Market	$7.749932 * 10^{-19}$	$1.204320e - 66$
Evolution Market	0	0
German Plaza Market	$9.276236 * 10^{-18}$	$1.049758 * 10^{-44}$
Hansa Market	$4.727538 * 10^{-159}$	0
Middle Earth Marketplace	$9.356239 * 10^{-20}$	$2.203038 * 10^{-83}$
Nucleus Market	$2.538463 * 10^{-174}$	$6.319438 *^{-268}$
Olympus Market	$1.453169 * 10^{-03}$	$1.647657 * 10^{-22}$
Pandora OpenMarket	$5.903384 * 10^{-65}$	$1.622666 * 10^{-187}$
Russian Anonymous Marketplace	$3.544511 * 10^{-83}$	$1.673279 * 10^{-48}$
Sheep Marketplace	$4.899846 * 10^{-112}$	$2.234014 * 10^{-231}$
Silk Road 2 Market	0	0
Silk Road Marketplace	0	0
TradeRoute Market	$1.563685 * 10^{-63}$	$3.078314 * 10^{-166}$
Wall Street Market	$8.111283 * 10^{-56}$	$1.109606 * 10^{-123}$

Table S3: *P* **values between the migrants and stayers** The table shows the *p* value results from the Kolmogorov smirnov test between the migrant and non migrants users distributions. The table report the results for the transactions to/from dark markets and the results for all the transactions.

References

[1] Dorit Ron and Adi Shamir. Quantitative analysis of the full bitcoin transaction graph. In *International Conference on Financial Cryptography and Data Security*, pages 6–24. Springer, 2013.

[2] Sarah Meiklejohn, Marjori Pomarole, Grant Jordan, Kirill Levchenko, Damon McCoy, Geoffrey M Voelker, and Stefan Savage. A fistful of bitcoins: characterizing payments among men with no names. In *Proceedings of the 2013 conference on Internet measurement conference*, pages 127–140. ACM, 2013.

[3] Elli Androulaki, Ghassan O Karame, Marc Roeschlin, Tobias Scherer, and Srdjan Capkun. Evaluating user privacy in bitcoin. In *International Conference on Financial Cryptography and Data Security*, pages 34–51. Springer, 2013.

[4] YoonJae Chung. Cracking the code: How the us government tracks bitcoin transactions. *ANALYSIS OF APPLIED MATHEMATICS*, page 152, 2019.

[5] Julia Heidemann, Mathias Klier, and Florian Probst. Identifying key users in online social networks: A pagerank based approach. 2010.

[6] Michael Fleder, Michael S Kester, and Sudeep Pillai. Bitcoin transaction graph analysis. *arXiv preprint arXiv:1502.01657*, 2015.

[7] Wikipedia Now Accepts Bitcoin Donations. `https://www.coindesk.com/wikipedia-now-accepts-bitcoin-donations`, 2019. Accessed: 1 October 2019.

[8] Chainalysis, inc. https://www.chainalysis.com/, 2019. Accessed: 10 July 2019.
